# Quantitative Trait Loci and Candidate Genes Associated with Photoperiod Sensitivity in Lettuce (*Lactuca spp.*)

**DOI:** 10.1007/s00122-021-03908-w

**Published:** 2021-07-10

**Authors:** Rongkui Han, Dean Lavelle, Maria José Truco, Richard Michelmore

**Affiliations:** 1 The Plant Biology Graduate Group, University of California, Davis, 95616 USA; 2 The Genome Center, University of California, Davis, 95616 USA; 3 Department of Plant Sciences, University of California, Davis, 95616 USA

## Abstract

**Key message:**

A population of lettuce that segregated for photoperiod sensitivity was planted under long-day and short-day conditions. Genetic mapping revealed two distinct sets of QTLs controlling daylength-independent and photoperiod-sensitive flowering time.

**Abstract:**

The molecular mechanism of flowering time regulation in lettuce is of interest to both geneticists and breeders because of the extensive impact of this trait on agricultural production. Lettuce is a facultative long-day plant which changes in flowering time in response to photoperiod. Variations exist in both flowering time and the degree of photoperiod sensitivity among accessions of wild (*Lactuca serriola*) and cultivated (*L. sativa*) lettuce. An F_6_ population of 236 recombinant inbred lines (RILs) was previously developed from a cross between a late-flowering, photoperiod-sensitive *L. serriola* accession and an early-flowering, photoperiod-insensitive *L. sativa* accession. This population was planted under long-day (LD) and short-day (SD) conditions in a total of four field and screenhouse trials; the developmental phenotype was scored weekly in each trial. Using genotyping-by-sequencing (GBS) data of the RILs, quantitative trait loci (QTL) mapping revealed five flowering time QTLs that together explained more than 20% of the variation in flowering time under LD conditions. Using two independent statistical models to extract the photoperiod sensitivity phenotype from the LD and SD flowering time data, we identified an additional five QTLs that together explained more than 30% of the variation in photoperiod sensitivity in the population. Orthology and sequence analysis of genes within the nine QTLs revealed potential functional equivalents in the lettuce genome to the key regulators of flowering time and photoperiodism, *FD* and *CONSTANS*, respectively, in Arabidopsis.

**Supplementary Information:**

The online version contains supplementary material available at 10.1007/s00122-021-03908-w.

## Introduction

Flowering time is a complex phenotype affected by both the genetic makeup of the plant and environmental conditions (Srikanth and Schmid 2011). Environmental factors documented to influence flowering time include vernalization (Johanson et al. 2000; Gendall et al. 2001; Searle et al. 2006), ambient temperature (Balasubramanian et al. 2006; Reeves et al. 2007), daylength (Putterill et al. 1995; Searle and Coupland 2004), and light quality and intensity, due to their impact on accumulation of photosynthates (Cho et al. 2018). The control of floral initiation by daylength is termed photoperiodism. Daylength is a key indicator of seasonality; hence, it is an important environmental variable that determines the timing of a plant’s transition to reproductive growth (Lang 1965). *Arabidopsis thaliana* is a facultative long-day (LD) plant, for which longer photoperiods accelerate flowering in an incremental fashion, while shorter photoperiods delay flowering but do not completely suppress it (Mouradov et al. 2002; Fornara et al. 2010). The current model for the molecular mechanism of the photoperiodic control of flowering time in Arabidopsis centers around a zinc finger transcription factor, *CONSTANS* (*CO*). Arabidopsis alters flowering time as a result of differential accumulation of CO in its leaf tissues under different daylengths. A multi-layer regulatory network controls the quantity of CO by regulating the transcription level of the *CO* gene (Park et al. 1999; An et al. 2004; Imaizumi et al. 2005) and by coordinating ubiquitin-mediated post-translational degradation of its protein product in a phytochrome- and circadian-clock-dependent fashion (Jang et al. 2008). Given this regulatory network, CO accumulates more readily under LD conditions (Suárez-López et al. 2001). This subsequently promotes the expression of *FLOWERING LOCUS T* (*FT*) in leaf tissues, from where the protein and mRNA encoded by *FT* travel up the vascular system through phloem companion cells and transduce the floral initiation signal to the shoot apical meristem (Corbesier et al. 2007; Li et al. 2011).

Wild lettuce (*Lactuca serriola*), the wild progenitor of cultivated lettuce (*L. sativa*; Kesseli et al. 1991), is a facultative LD plant (Sukprakarn 1985). Cultivated lettuce also exhibits varying degrees of responsiveness to photoperiod, although its sensitivity is generally muted in comparison to its wild relative. Some cultivars (cvs.), such as the North American crisphead “Empire” and “Salinas,” are less sensitive to photoperiod than others, such as the European butterheads “May King” and “Saffier,” for which flowering time is significantly delayed as the daylength shortens (Waycott 1995). Lettuce is harvested for its vegetative tissues for consumption as a leafy vegetable in the western diet and for its enlarged vegetative stem in East Asian cuisines (Zhang et al. 2017). Floral initiation in lettuce is accompanied by the increase of bitter flavors (Ryder 1996), which drastically diminishes the culinary quality of the vegetable. As a result, delayed flowering is a major consideration in lettuce breeding to maximize harvestable yield (Thompson and Ryder 1961). Because the flowering time trait exhibits photoperiod dependency, it is also desirable to breed for lettuce with stable flowering time across different growing areas and daylength conditions. Understanding the genetics and molecular mechanism of the photoperiodic regulation of flowering time in lettuce can enhance the efficiency of breeding endeavors.

In the past decade, multiple genetic mapping and association studies have reported genetic loci controlling bolting (Lavelle 2009; Jenni et al. 2013; Mamo et al. 2019; Sthapit Kandel et al. 2020; Seki et al. 2020) and flowering time traits in lettuce (Hartman et al. 2012, 2013a, b; Kwon et al. 2013; Niroula 2017). A recent review on this topic reported a total of 64 quantitative trait loci (QTLs) associated with bolting and/or flowering time phenotypes in lettuce (Han et al. 2021a). Two QTLs on Chromosomes 2 and 7, *qFLT2.1* and *qFLT7.1*, have shown major effects on lettuce flowering time across multiple mapping populations. There have been fewer molecular studies on flowering time. No homolog or functional equivalent of *CO* has been identified in lettuce (Lavelle 2009; Abbott 2010; Han et al. 2021a). A clone of the lettuce ortholog of Arabidopsis *FT*, “*LsFT*” (Lsat_1_v5_gn_2_17881) induced early flowering when ectopically expressed in transgenic Arabidopsis (Fukuda et al. 2011). Notably, *qFLT2.1* co-locates with *LsFT* (Han et al. 2021a).

Past studies have only examined bolting and/or flowering time under single photoperiod conditions and the vast majority have been conducted under only LD conditions (Han et al. 2021a). The genetics underlying the response of lettuce to changing photoperiods has not been investigated. This study exploited available genetic and genomic resources in lettuce to distinguish the photoperiodic response phenotype from the daylength-independent flowering time phenotype. A population of F_6_ recombinant inbred lines (RILs) was previously developed from a cross between a late-flowering, photoperiod-sensitive accession of *L. serriola*, Armenian999, and an early-flowering, photoperiod-insensitive landrace line of *L. sativa*, PI251246 (Sandoya et al. 2020). The population was planted under LD and short-day (SD) conditions in multiple environments. We partitioned the flowering time phenotype into its two components: daylength-independent flowering time (FLT) and photoperiodic sensitivity (PPS). Separate QTLs were identified for FLT and for PPS. The QTLs for these two traits did not co-locate, indicating separate genetic determinants controlling FLT and PPS.

## Materials and methods

### Plant material

A population of 236 F_6_ RILs was previously developed by single-seed descent from a cross between the *L. serriola* accession Armenian999 and the *L. sativa* landrace line PI251246 (Sandoya et al. 2020). Armenian999 flowers slightly later than PI251246 under LD conditions and exhibits strong sensitivity to photoperiod. PI251246 exhibits low sensitivity to photoperiod and therefore flowering is not delayed as much as Armenian999 under SD conditions. This population is suitable for studying flowering phenotypes because both parents do not form heads, which facilitates accurate scoring of floral initiation.

### Nightbreak experiment

The parental lines, Armenian999 and PI251246, were seeded on December 20th, 2019 and grown in a greenhouse at Davis, CA. Individual plants were grown in one-liter pots spaced one foot apart. Six plants of each line were randomized and grown on the same bench under 10 h/14 h light/dark cycles, while another six of each were randomized and grown in the same greenhouse on a separate bench with an additional nightbreak treatment. For the nightbreak treatment, the bench used for the treatment was surrounded by a non-light-permeable white plastic tarp; one hour of supplementary lighting was given from a high-pressure sodium growth light in the middle of the dark period every day. The time at which the first flower bud became visible was recorded for each plant. A photograph of one representative plant of each line from each treatment was taken on February 17th, 2019 using a Canon EOS 50D DSLR Camera.

### Growing conditions

The 236 RILs, both parental lines, and two controls, *L. sativa* cv. Salinas and *L. serriola* accession US96UC23 were planted in complete randomized blocks at two separate field locations in Davis and Salinas, California in summer 2019 to characterize flowering time under LD conditions. The same lines were planted in a field in Holtville, California and in a screenhouse without supplementary lighting in Davis, California in winter 2019–2020 (November–March) to characterize flowering time under SD conditions. All plants were seeded into 16 × 8 cell trays and grown into seedlings in the UC Davis Vegetable Crop Greenhouses facility. At 4–6 weeks old, seedlings were transplanted into the field (LD-Davis, LD-Salinas, and SD-Holtville) or one-gallon pots (SD-screenhouse). For field experiments, pre-plant N-P-K fertilizer, pre-emergence herbicide Balan DF, and post-emergence herbicide Kerb 50 W were applied to the fields at levels recommended by their respective labels. Details of the designs and plants of the experiments are shown in Table 1.

**Table 1:** Design and planting information for four experiments in 2019

Photoperiod	Location	Growth condition	Date of transplanting (MM/DD/YYYY)	RILs	Block	Plants per block
LD (Avg 14.39 h)	Davis, CA	Field	05/08/2019	236	2	6
LD (Avg 13.80 h)	Salinas, CA	Field	06/05/2019	236	2	8
SD (Avg 10.93 h)	Holtville, CA	Field	11/07/2019	236	2	8
SD (Avg 11.54 h)	Davis, CA	Screenhouse	12/17/2019	236	3	1

### Phenotyping

The developmental stage of individual plants was scored weekly, starting two weeks after transplanting. Flowering time of an individual was quantified as the time at which the first flower bud became visible. The average flowering time was calculated for each plot. Flowering time in days was transformed into growing degree days (GDDs). The number of GDDs accumulated in a given day, *d*, is calculated using the following formula:

$${\text{GDD}}_{d} \, = \,{\text{T}}_{{{\text{mean, d}}}} {-}{\text{T}}_{{{\text{base}}}}$$

This allowed for adjustment of the effect of temperature on flowering time, with T_*base*_ = 5.5 °C (Maynard 2014). Flowering time in units of GDDs was used as the phenotype for QTL mapping. The average temperature of each day, T_*mean, d*_, was calculated in Celsius from hourly measurements collected from the National Centers for Environmental Information website (https://www.ncdc.noaa.gov/) for the UC Davis University Airport, CA (Station ID WBAN:00174, GPS coordinates 38.533°, −121.783°), Salinas Airport, CA (Station ID: WBAN:23233, GPS coordinates 36.6636°, −121.6081°), and Imperial Co. Airport, CA (Station ID: WBAN:03144, GPS coordinates 32.83417°, −115.57861°) weather stations during the respective periods of the experiments. The weather stations in Davis, Salinas, and Imperial were 0.5 km, 7.1 km, and 12.7 km from the experimental plots, respectively.

Phenotypic values of photoperiod sensitivity (PPS) of the RILs were estimated separately for each SD experiment. PPS was calculated by subtracting the mean LD flowering time, averaged between the LD-Davis and LD-Salinas experiments, from the SD flowering time in each SD experiment:

$${\text{PPS}}_{{{\text{Holtville}}}} \, = \,{\text{SD}}_{{{\text{Holtville}}}} {-}{\text{LD}}_{{{\text{mean}}}}$$

$${\text{PPS}}_{{{\text{Screenhouse}}}} \, = \,{\text{SD}}_{{{\text{Screenhouse}}}} {-}{\text{LD}}_{{{\text{mean}}}}$$

This method provides biologically interpretable quantifications of photoperiodic responses (“number of GDDs the flowering time was delayed due to SD conditions”). It has been used in studies of photoperiodism in multiple plant species, including rice (Maheswaran et al. 2000), wheat (Sourdille et al. 2000), soybean (Tasma et al. 2001), maize (Coles et al. 2010), and Arabidopsis (Méndez-Vigo et al. 2013).

A linear modeling method was used as an alternative measure of photoperiod sensitivity to provide independent validation for the quantification method described above. The SD phenotype data of all RILs from the Holtville field trial and the screenhouse trial were separately regressed with their genotype on the major LD flowering time QTL *qFLT4.1* using ordinary least square regression. The residuals of the regression were used as quantifications of photoperiod sensitivity:

$${\text{SD}}_{{{\text{Holtville}}}} = ~\hat{\beta }_{{_{{{\text{Holtville}}}} }} \, \times \,{\text{qFLT4}}.{\text{1}}_{{\{ 0,{\text{1}}\} }} \, + \,{\text{PPS}}^\prime_{{{\text{Holtville}}}} .$$

$${\text{SD}}_{{{\text{Screenhouse}}}} = ~\hat{\beta }_{{_{{{\text{Screenhouse}}}} }} \, \times \,{\text{qFLT4}}.{\text{1}}_{{\{ 0,{\text{1}}\} }} \, + \,{\text{PPS}}^\prime_{{{\text{Screenhouse}}}} .$$

This method of isolating the photoperiod sensitivity component of flowering time may produce less biologically interpretable measurements; nevertheless, it ensures numeric independence of the photoperiodic component and the LD flowering time component.

### Genotyping and QTL analysis

Parental lines and RILs were subjected to genotyping-by-sequencing using 100 bp paired-end Illumina HiSeq 4000 as described in (Han et al. 2021b). The parental lines, Armenian999 and PI251246, were also whole-genome-shotgun sequenced using 150 bp and 100 bp paired-end Illumina HiSeq4000 to 29 × and 17 × coverages, respectively. Sequencing results were mapped to version 8 of the lettuce reference assembly (Reyes-Chin-Wo et al. 2017; NCBI: GCA_002870075.2) using bwa-mem (Li 2013). High-confidence single nucleotide polymorphism (SNP) markers were identified using the software FreeBayes (Garrison and Marth 2012). A genetic map was constructed using the software LepMap3 (Rastas 2017). Detailed protocols for genotyping and genetic map construction are described in Han et al. (2021b). Heritability of the phenotypes was estimated using mixed effect modeling with R packages “synbreed” (Wimmer et al. 2012) and “sommer” (Covarrubias-Pazaran 2016), using location as fixed effect and marker-estimated genetic relationship as random effect. QTL analysis was performed using 2677 high-quality SNP markers, each representing one distinct genetic bin. Composite interval mapping was performed using the R package “qtl” (Broman et al. 2003) to determine QTL peaks, intervals, and effects. The significance threshold was determined using a 1000-iteration permutation test with *p* < 0.05. The region within 1-log-of-odds (LOD) score of each locus with a local peak LOD score above the significant threshold was extracted as a QTL interval. The QTLs were named using an acronym of the phenotype (“FLT” for LD flowering time, “PPS” for photoperiod sensitivity), the chromosomal location of the QTL, and a number that reflects the order of discovery of the QTL after taking into account previously reported flowering time QTLs (Han et al. 2021a).

### Genomic analysis

Lettuce orthologs of flowering-time genes in Arabidopsis were identified as follows: proteome of seven eudicot species, *Arabidopsis thaliana*, *Solanum lycopersicum*, *Daucus carota*, *Cynara cardunculus*, *Helianthus annuus*, *L. serriola*, and *L. saliva*, were clustered into orthology groups using software Orthofinder (Emms and Kelly 2015); lettuce genes within the same orthology groups as Arabidopsis flowering time genes were then extracted and located in the reference genome. Amino acid sequences within the orthology groups of Arabidopsis *FD*, Phytochromes and *CONSTANS*, were aligned using ClustalOmega (Madeira et al. 2019). The circadian expression profile of the putative orthologs was as described in Supplementary Table 3 of Han et al. (2021a). Single nucleotide variants, insertions, deletions, stop-loss variants, and stop-gain variants were identified between the parental lines using the software ANNOVAR (Wang et al. 2010).

## Results

### LD phenotype and daylength-independent flowering time QTL

**Fig. 1:**
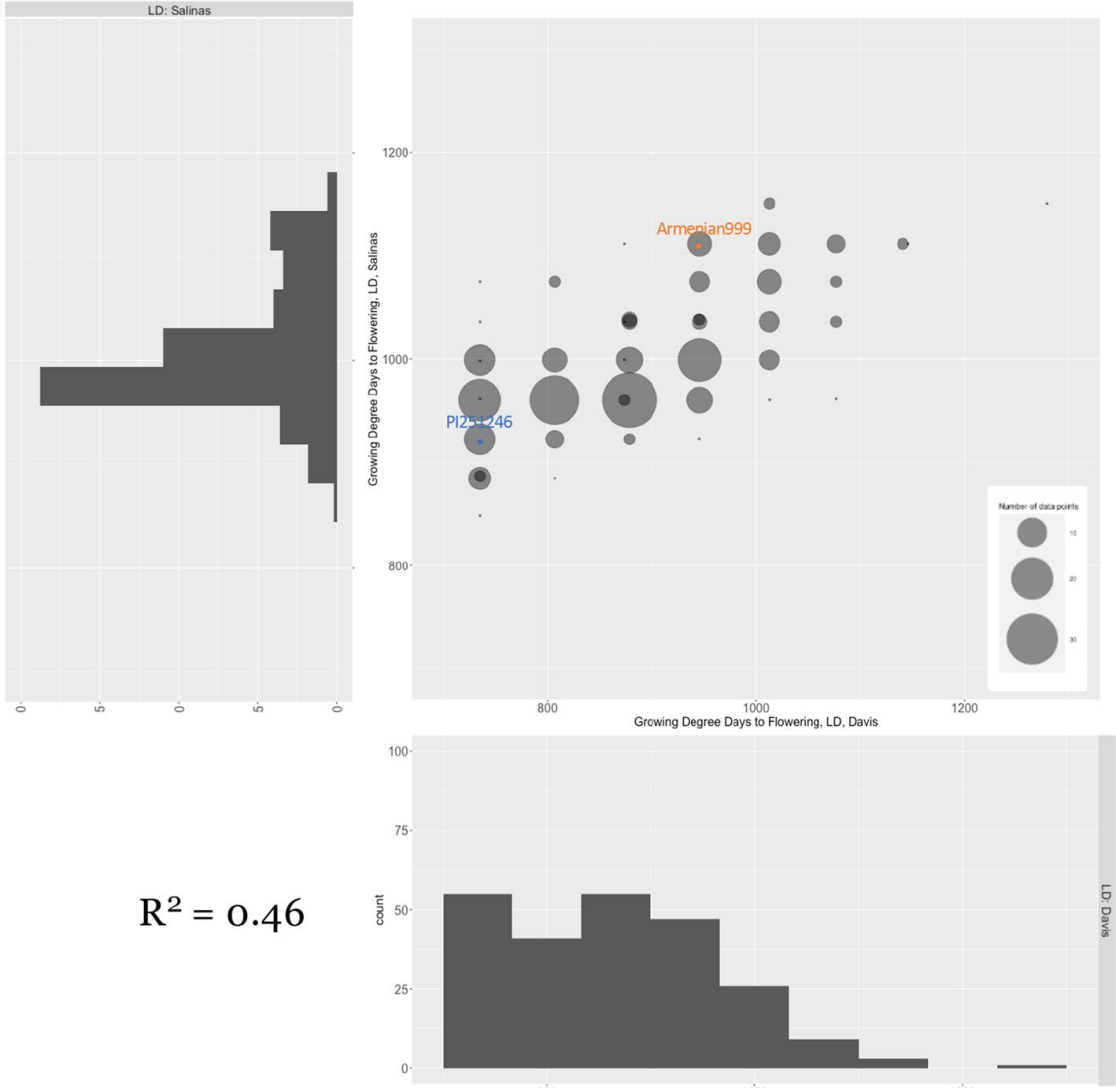
Correlation in long-day flowering time of the mapping population in two environments. The long-day flowering time of the Armenian999 (*L. serriola*) × PI251246 (*L. sativa*) F_6_ recombinant inbred line (RIL) population was scored in weekly intervals in two field experiments in Salinas and Davis, CA. The dot plot presents the phenotype of the same RILs in the two experiments (x-axis: Davis; y-axis: Salinas). Flowering time is expressed in growing degree days

The LD flowering time phenotype, measured in GDDs after planting (GAP), showed right-skewed Gaussian-like distribution in both the Salinas and Davis experiments (Fig. 1). The phenotypic distribution was truncated at the lower end in the Davis experiment because the earliest line flowered before the first occurrence of phenotyping. The difference between the earliest flowering event in the experiment and the time of first phenotyping was estimated to be less than a week, given the condition of the plants at the time of phenotyping. To avoid introducing bias, this error was not manually corrected. Phenotypic values collected from the two LD experiments showed a significant correlation with each other (*R*^2^ = 0.46, *p* < 10^–16^; Fig. 1). Plants in the Davis experiment showed overall earlier flowering. The mean flowering time in Davis was 120 GAPs earlier than in Salinas. Under LD conditions, the insensitive parent (PI251246) flowered at 828.6 GAP on average, while the sensitive parent (Armenian999) had an average flowering time of 1028.6 GAP (Table 2, Rows 1 & 2). Transgressive segregation of LD flowering time was observed on the right (later flowering) end of the phenotypic distribution in the Davis trial and on both ends of the distribution in the Salinas trial (Fig. 1).

**Table 2:** Mean and range of values for flowering time, measured in growing degree days after planting, in a *L. serriola* × *L. sativa* F_6_ RIL population and parents in two long-day (LD) experiments conducted in summer 2019 (Rows 1 & 2), two short-day (SD) experiments conducted in winter 2019–2020 (Rows 3 & 4), and photoperiodic response, derived from subtracting mean LD flowering time from experiment-level SD flowering time (Rows 5 & 6). SE indicates standard error

Phenotype	Location	Parents		RILs			
		PI251246	Armenian999	Min	Max	Mean	SE
LD flowering time	Davis	734.9	945.6	734.9	1279.2	873.6	107.8
	Salinas	922.4	1111.5	848.6	1150.6	993.2	59.1
SD flowering time	Holtville	961.4	1927.9	1011	1928	1571	253.5
	Screenhouse	992.9	1556.5	952.1	1647.6	1230.2	159.2
Photoperiodic response	Holtville	132.8	899.3	192.2	1080.3	639.9	253.5
	Screenhouse	164.3	528.0	63.9	638.3	296.5	159.2

The genotype of the RILs at 2677 polymorphic SNP sites were used to construct a genetic map. The map covered 1883 cM in nine chromosomal linkage groups (LGs). The mean distance between each pair of adjacent markers was 0.7 cM. Three gaps between 5 and 7 cM are present in this map located at 149.0–155.8 cM on linkage group 3, 62.8–68.1 cM on linkage group 7, and 50.3–55.7 cM on linkage group 9. There was one gap of 10.4 cM on linkage group 3. Four RILs were excluded from downstream analyses due to the large percentage of missing genotype data, resulting in a final set of 232 RILs for QTL mapping (Han et al. 2021b).

**Fig. 2:**
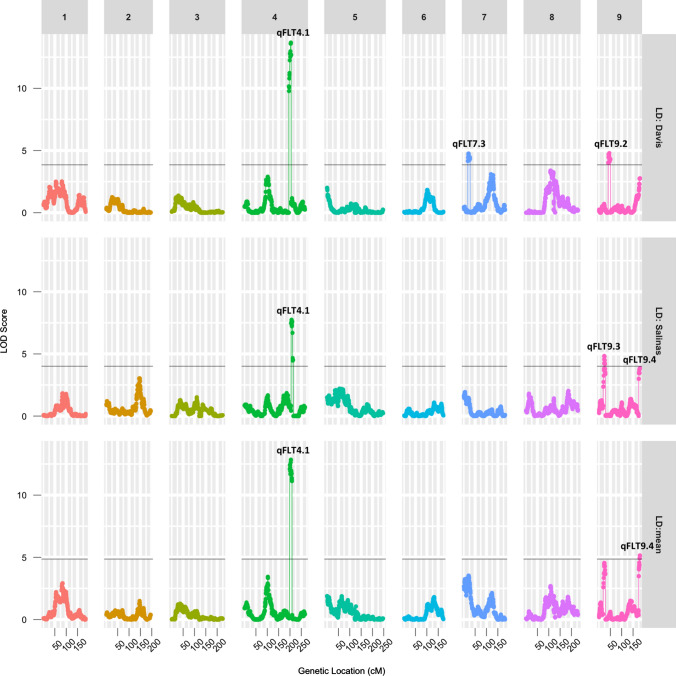
Logarithm of odds (LOD) scores of markers for long-day flowering time, shown along the nine chromosomal linkage groups. The LOD threshold for significance (*p* < 0.05) calculated by 1000 permutations is shown as a black line

Broad sense heritability of flowering time under LD conditions was estimated to be 0.72 using a mixed effect model. Composite interval mapping revealed five significant QTLs for LD flowering time on LGs 4, 7, and 9. The individual QTLs accounted for 4.95–18.82% of the phenotypic variation (Fig. 2; Table 3). Two of these QTLs, *qFLT4.1* and *qFLT9.4*, were captured with QTL mapping that was re-performed using phenotypic data averaged across the two locations (Table 3).

**Table 3:** A total of five QTLs, *qFLT4.1*, *7.3*, *9.2*, *9.3,* and *9.4*, were detected for LD flowering time in an Armenian999 × PI251246 F_6_ RIL population evaluated in two field experiments in summer 2019

Location	QTL	Chr	Marker closed to peak	Interval (cM)	LOD^‡^	PVE^*^	Allele^†^
Davis	*qFLT4.1*	4	Lsat_1_v8_lg_4.283169022_283617815	195.0–205.1	14.06	18.73	A
	*qFLT7.3*	7	Lsat_1_v8_lg_7.30591573_30591595	22.0–30.5	4.56	4.95	A
	*qFLT9.2*	9	Lsat_1_v8_lg_9.48974335_48974387	40.4–55.6	5.81	6.13	P
Salinas	*qFLT4.1*	4	Lsat_1_v8_lg_4.294973197_295012418	198.0–209.0	7.98	12.05	A
	*qFLT9.3*	9	Lsat_1_v8_lg_9.29591529_30108481	22–26.6	4.67	7.35	P
	*qFLT9.4*	9	Lsat_1_v8_lg_9.200741908_204015989	175.2–180.8	4.67	6.65	A
Mean	*qFLT4.1*	4	Lsat_1_v8_lg_4.283169022_283617815	198.0–206.0	13.01	18.9	A
	*qFLT9.4*	9	Lsat_1_v8_lg_9.200088730_204243350	176.9–180.8	5.06	6.51	A

^‡^: Log of odds

^*^: Percent variance explained

^**†**^: The parental allele that increased the trait value. “A” represents Armenian999 and “P” represents PI251246. The same abbreviations were used in all other tables

QTL *qFLT4.1* was detected in both experiments and had the highest LOD score among all LD flowering time QTLs. This QTL explained 12.05–18.73% of the variance in LD flowering time. The presence of the allele from the late-flowering parent Armenian999 on *qFLT4.1* delayed flowering. The allelic effect is reversed on *qFLT9.2* and *qFLT9.3*, where the allele from PI251246 contributed to delayed flowering. However, the effects of *qFLT9.2* and *qFLT9.3* were not significant when analyzing the mean phenotype across the two locations. No epistatic interactions were found between the QTLs.

### Nightbreak experiment

**Fig. 3:**
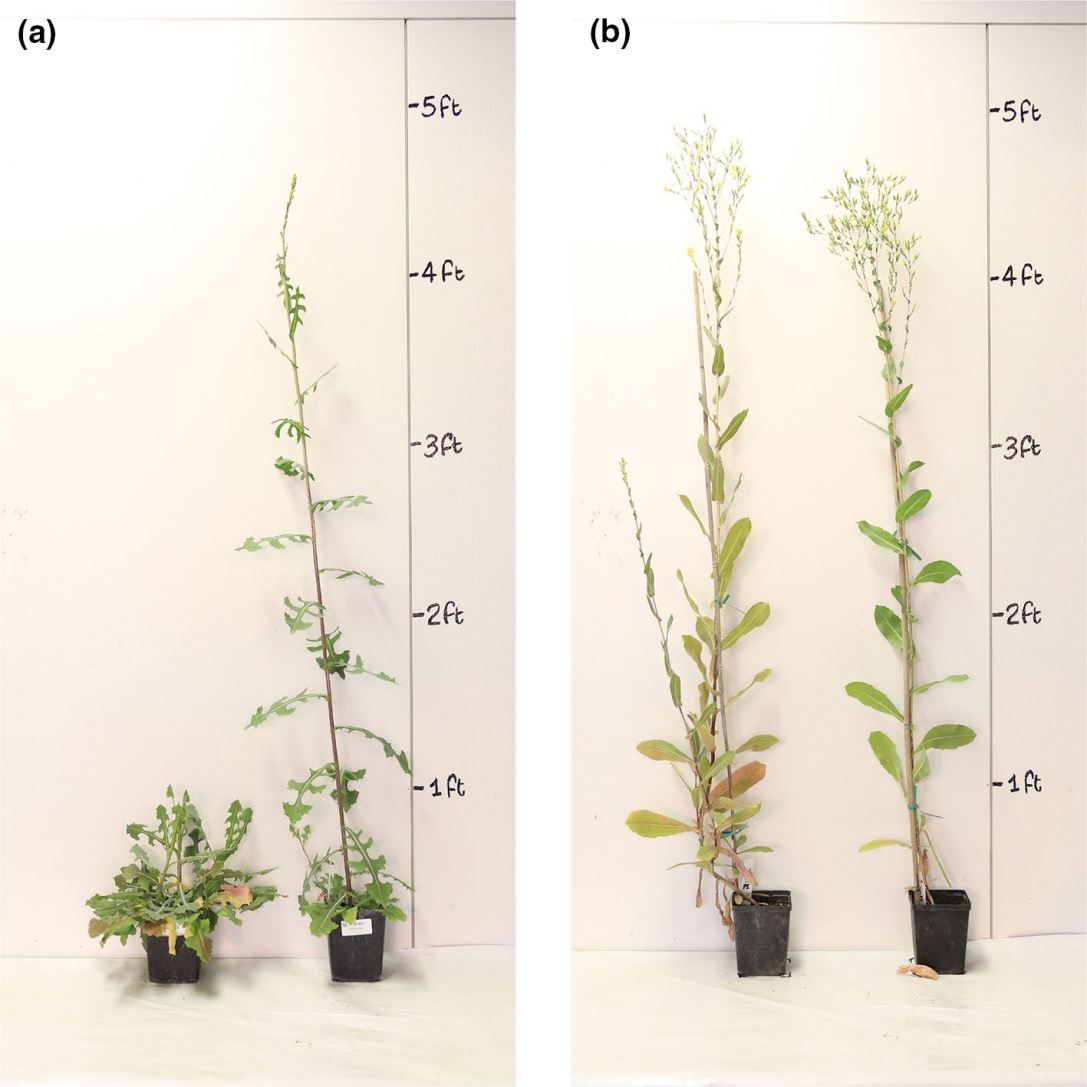
Contrasting responses of the parental lines to the nightbreak treatment. The plant on the left in each panel was grown for 61 days under 10 h/14 h light/dark cycles in a greenhouse; the plant on the right in each panel was grown in the same greenhouse with an additional 1-h nightbreak treatment implemented in the middle of the dark period. **a** Photoperiod sensitive parent, Armenian999. **b** Photoperiod insensitive parent, PI251246

The parents of the mapping population responded differently to one hour of light in the middle of the night (dark period) in an otherwise SD regime in the greenhouse (Fig. 3). The photoperiod-sensitive parent, Armenian999, flowered on average 78 days after planting (DAPs) in the control group and 57 DAPs in the nightbreak treatment; nightbreak significantly accelerated flowering by 21 days (*p* < 2 × 10^–16^). In contrast, the insensitive parent, PI251246, flowered on average 43 DAPs in the control group and 34.25 DAPs in the nightbreak group; nightbreak only accelerated flowering by 8.75 days and was not found to be significant (*p* = 0.12).

### Partitioning of photoperiod sensitivity component of the flowering time phenotype

**Fig. 4:**
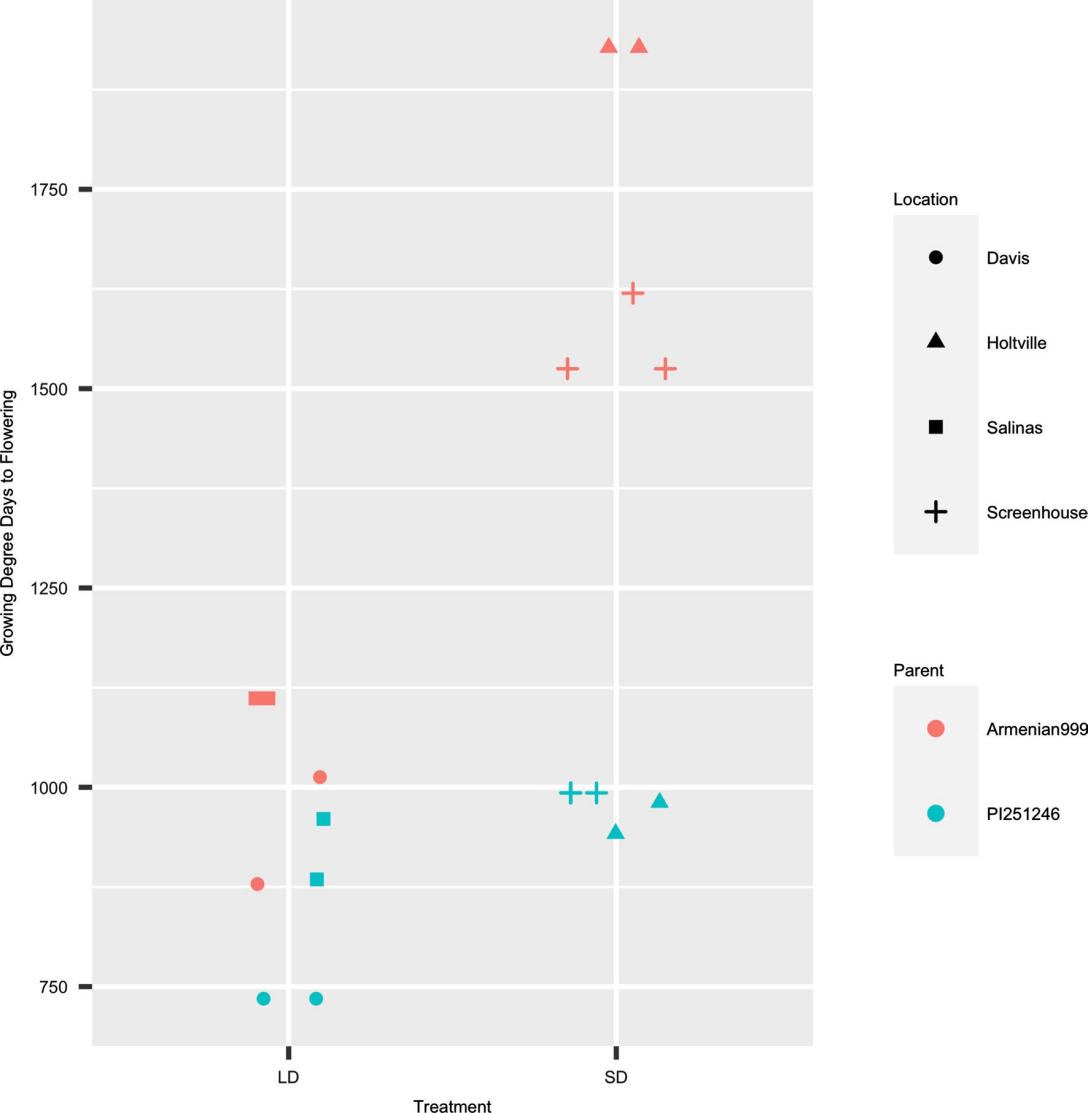
Flowering time (in growing degree days) of the parental lines, Armenian999 (*L. serriola*) and PI251246 (*L. sativa*) in both long-day (Salinas and Davis) and both short-day (Holtville and screenhouse) experiments. Armenian999 exhibited a strong photoperiodic response in flowering time, while PI251246 was insensitive

**Fig. 5:**
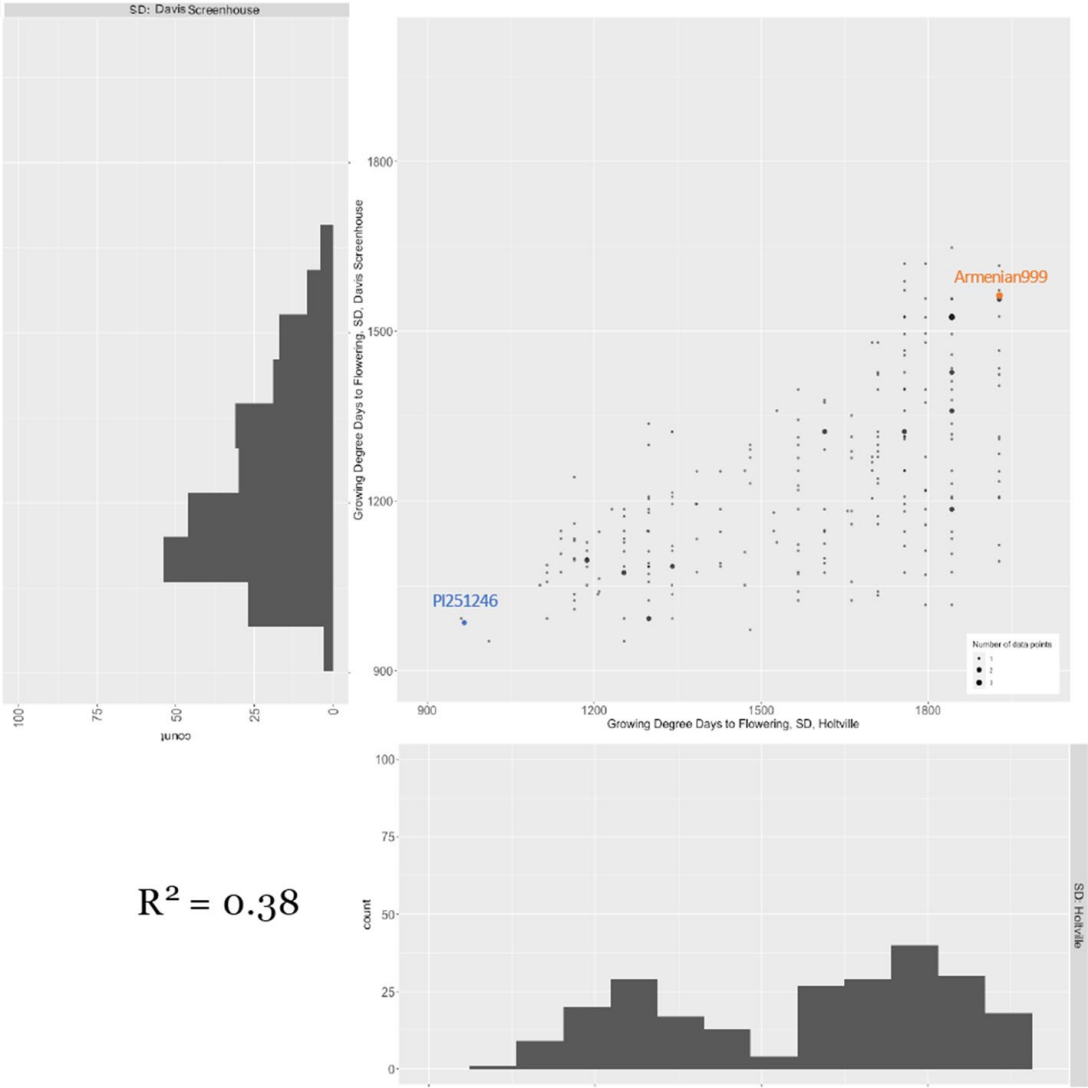
Phenotypic distributions of short-day flowering time of the Armenian999 (*L. serriola*) × PI251246 (*L. sativa*) F_6_ RIL population in two experiments conducted in an experimental field in Holtville, CA and a screenhouse in Davis, CA under short-day winter conditions. The dot plot presents the phenotype of the same RILs in the two experiments (x-axis: Holtville; y-axis: screenhouse). Flowering time is expressed in growing degree days

The genetics of sensitivity to daylength was examined by growing the same RIL population under SD conditions in the field and in a screenhouse with no supplementary lighting. Comparison of plot-level SD and LD flowering time data (in GAP) revealed that, under short photoperiod conditions, flowering was barely delayed in the insensitive parent PI251246 (*p* = 0.11) but significantly delayed in Armenian999 (*p* = 2.56 × 10^–6^; Fig. 4). On a population level, the LD flowering time of a RIL is a poor predictor of its SD flowering time; the average pairwise coefficient of determination (*R*^2^) is 0.33 for any pair of LD-SD experiments. This indicates that, in addition to daylength-independent flowering time regulation, there are separate genetic mechanisms for photoperiodic regulation of reproductive growth. In addition, the SD flowering time phenotype exhibited higher location-sensitivity than the LD flowering time phenotype. The SD flowering time phenotype had lower, although still significant, correlation between experiments (*R*^2^ = 0.38, *p* < 10^–16^), with the mean SD flowering time 339.4 GAPs later in the Holtville experiment in the Imperial Valley than in the screenhouse experiment at Davis (Table 2, Rows 3 & 4; Fig. 5). The phenotype followed a right-skewed Gaussian-like distribution in the screenhouse experiment; in the Holtville field experiment, the phenotype showed clear bimodal distribution, with two peaks at 1300 and 1800 GDDs (Fig. 5). The bimodal distribution of the phenotype in the Holtville experiment is consistent with the presence of at least one major genetic locus that control daylength sensitivity. Broad sense heritability of flowering time under SD conditions was estimated to be 0.89 using a mixed effect model.

Phenotypic values of PPS of the RILs in each SD experiment were calculated by subtracting the mean LD flowering time from the SD flowering time in each SD experiment. Flowering of the sensitive parent, Armenian999, was significantly delayed as it flowered 899.3 (Holtville) and 528.0 (screenhouse) GDDs later in SD conditions than in LD conditions. In contrast, flowering of the insensitive parent PI251246 was delayed much less as it flowered 132.8 (Holtville) and 164.3 (screenhouse) GDDs later (Table 2, Rows 5 & 6). There was no strong evidence of transgressive segregation of photoperiod sensitivity. Overall, the photoperiodic response was stronger in the field at Holtville than in the Screenhouse at Davis. A linear model was used as a second method to validate the results of the genetic analysis of PPS (PPS´, see Material and Methods). Across both SD experiments, PPS and PPS´ were strongly correlated (R^2^_Holtville_ = 0.89, R^2^_screenhouse_ = 0.80; Fig. 5).

### Photoperiod sensitivity QTL

**Fig. 6:**
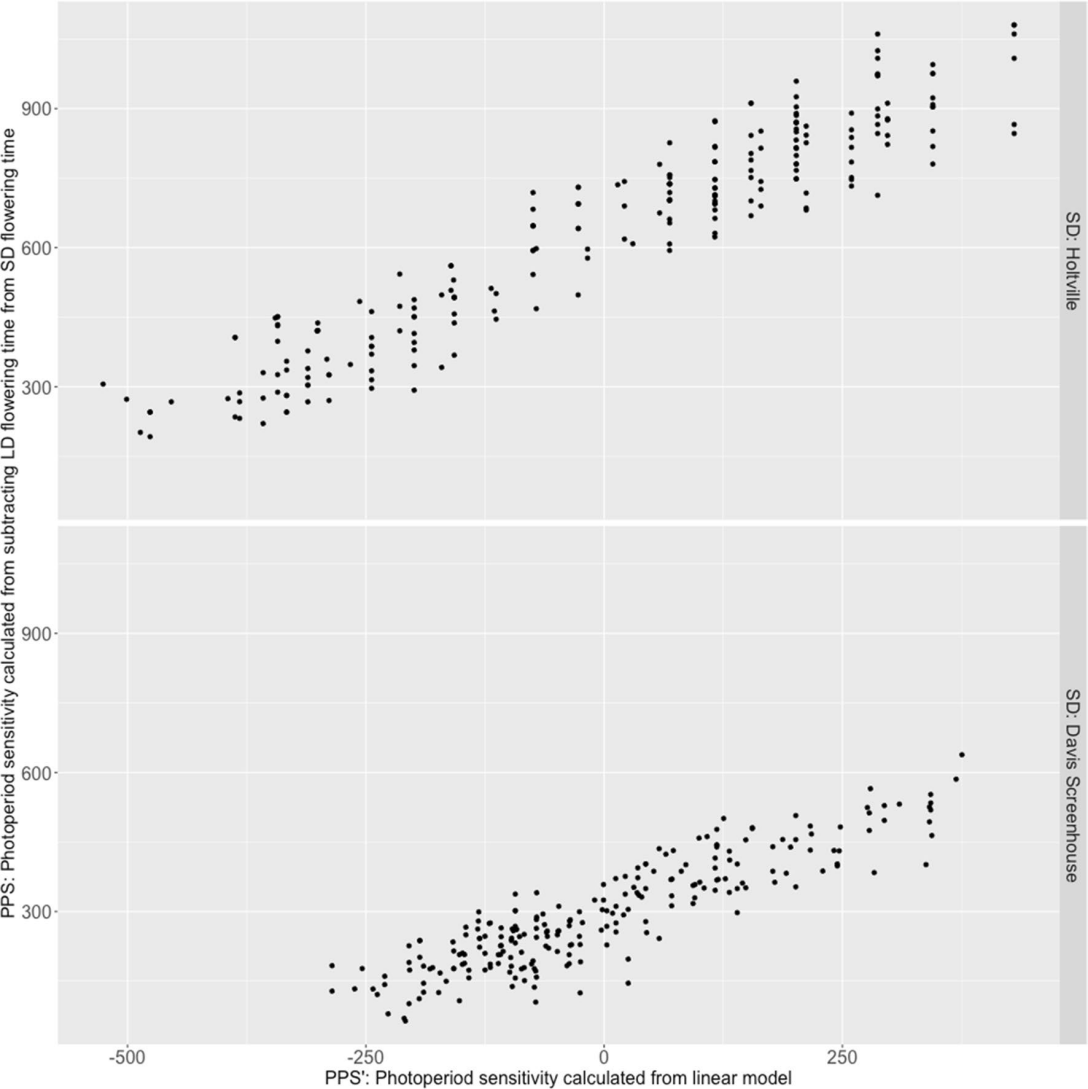
Strong correlation between results of two methods of quantifying photoperiod sensitivity (PPS)

**Fig. 7:**
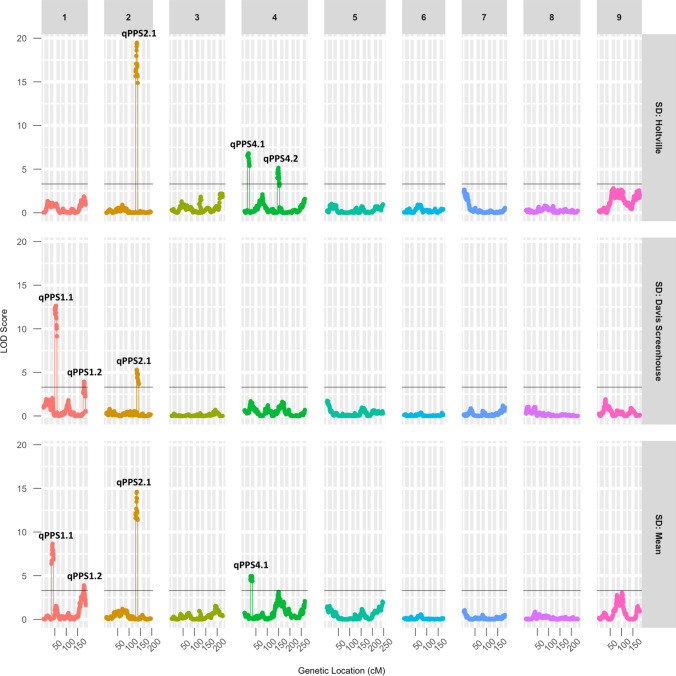
LOD scores of markers for photoperiod sensitivity shown along the nine chromosomal linkage groups. The LOD threshold for significance (*p* < 0.05) calculated by 1000 permutations is shown as a black line

Composite interval mapping revealed five significant QTLs for photoperiod sensitivity on LGs 1, 2, and 4. Individual QTLs accounted for 5.59–28.98% of the phenotypic variation (Table 4; Fig. 6). There are no overlaps between LD flowering time QTLs and PPS QTLs (Fig. 7).

**Table 4:** A total of five QTLs were detected for photoperiod sensitivity in a *L. serriola* × *L. sativa* F_6_ RIL population evaluated in two long-day experiments and two short-day experiments in summer 2019 and winter 2019–2020

Location	QTL	Chr	Marker closed to peak	Interval (cM)	LOD	PVE	Allele^*^
Holtville	*qPPS2.1*	2	Lsat_1_v8_lg_2.171422510_171778602	132.0–135.8	19.77	28.98	A
	*qPPS4.1*	4	Lsat_1_v8_lg_4.33492617_34012680	12.4–20.6	6.39	10.27	A
	*qPPS4.2*	4	Lsat_1_v8_lg_4.192761242_193410678	145.0–151.5	5.50	9.13	A
Screenhouse	*qPPS1.1*	1	Lsat_1_v8_lg_1.46475164_46488701	49–55.4	12.84	20.07	A
	*qPPS1.2*	1	Lsat_1_v8_lg_1.189492341_201862756	175.0–182.0	4.51	6.05	A
	*qPPS2.1*	2	Lsat_1_v8_lg_2.170127374_170849299	128.6–138.8	5.93	7.39	A
Mean	*qPPS1.1*	1	Lsat_1_v8_lg_1.50417917_50417917	49.0–60.0	5.71	6.82	A
	*qPPS1.2*	1	Lsat_1_v8_lg_1.190901262_2027901202	176.0–181.1	4.41	5.59	A
	*qPPS2.1*	2	Lsat_1_v8_lg_2.170127374_170849299	132.0–135.8	17.28	26.65	A
	*qPPS4.1*	4	Lsat_1_v8_lg_4.43189585_43189629	22.0–33.0	5.69	8.64	A

^*^: The parental allele that increased the trait value

The presence of the allele from the photoperiod-sensitive parent, Armenian999, on all PPS QTLs delayed flowering under SD conditions. QTL *qPPS2.1* was detected in both experiments and had a very large effect in the Holtville experiment. No significant epistatic interactions were detected between any pair of QTLs. Together, these QTLs explained 44.5% of variance in photoperiod sensitivity in the Holtville experiment and 33.45% in the screenhouse experiment.

The result of the QTL analysis was confirmed using an independent data analysis protocol that calculated PPS by regressing SD phenotype using the genotype of the major LD flowering time QTLs *qFLT4.1* as fixed effect (Fig. 7). The results of the two independent analyses strongly agree with each other (*p* < 10^–16^; Supp. Figure 1). The three most consistent and largest-effect PPS QLTs, *qPPS1.1*, *2.1*, and *4.1*, were discovered using both methods (Supp. Table 1a). QTL analysis was also performed using just the SD flowering time data. This revealed the same three QTLs (*qPPS1.1*, *2.1* and *4.1*) plus *qFLT4.1,* which corresponds to the locus controlling flowering time under both LD and SD conditions (Supp. Table 1b; Supp. Figure 2). The comparison of LD, PPS, and SD QTLs is presented in Supp. Figure 3.

### Candidate genes

**Fig. 8:**
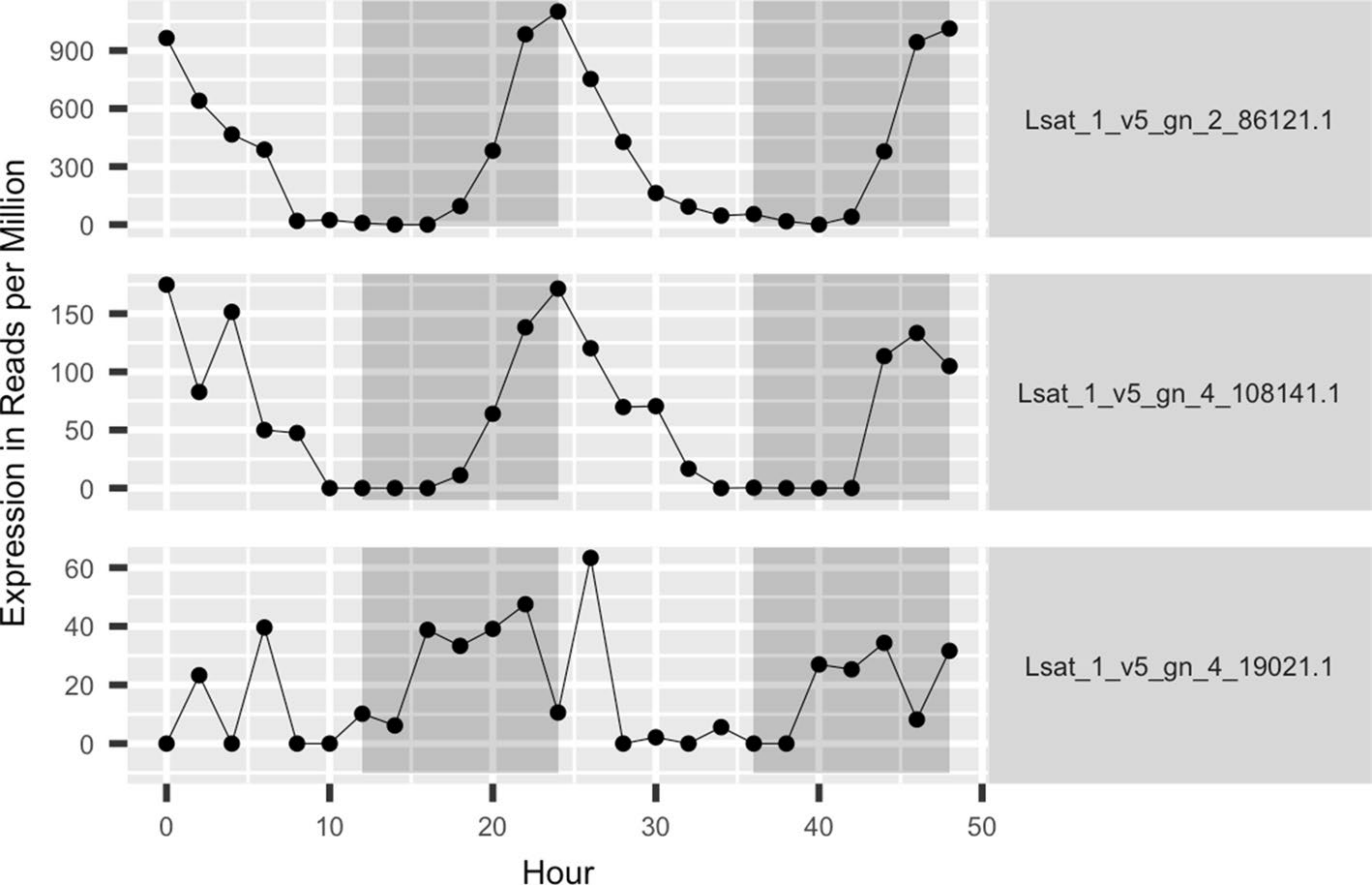
Forty-eight-hour expression profiles of three candidate genes in QTLs *qPPS2.1* (Lsat_1_v5_gn_2_86121, orthologous to *AtCO*), *qPPS4.1* (Lsat_1_v5_gn_4_19021, orthologous to At*PFT1*), and *qPPS4.2* (Lsat_1_v5_gn_4_108141, orthologous to *AtCDF*) in vegetative lettuce leaf tissues. Time-series RNA-seq data was obtained from the 12 h light/12 h dark experiment described in Higashi et al. (2016). Expression was quantified in reads per million mapped reads

All of the detected FLT and PPS QTLs, except for *qPPS1.2*, contain genes that are orthologous to genes with flowering time related functions in Arabidopsis. Forty-five of these genes harbor non-synonymous variants between the parental lines within their coding sequences (Supplementary Table 2). Six of these genes show a rhythmically oscillating expression pattern during a day–night cycle in vegetative lettuce leaf tissues (Supplementary Table 2; Higashi et al. 2016). Among them, three have orthologs that function in the photoperiod pathway in Arabidopsis, including Lsat_1_v5_gn_2_86121 (orthologous to *CO*; Putterill et al. 1995) in *qPPS2.1*, Lsat_1_v5_gn_4_19021 (orthologous to *PFT1*; Iñigo et al. 2012) in *qPPS4.1*, and Lsat_1_v5_gn_4_108141 (orthologous to *CDF1*; Imaizumi et al. 2005) in *qPPS4.2*. The 48-h expression profile of these three genes in vegetative lettuce leaf tissue grown under 12 h/12 h light/dark cycles is shown in Fig. 8. A detailed summary of all non-synonymous sequence variants within candidate genes for all QTLs are listed in Supplementary Table 2.

## Discussion

In this study, a lettuce RIL population that segregated for both daylength-independent flowering time and photoperiod sensitivity was grown under multiple daylength conditions. QTL analysis revealed that these two phenotypes were determined by different loci. Both flowering time and photoperiod sensitivity showed high heritability. Photoperiod sensitivity exhibited higher variation across different environments than daylength-independent flowering time. We identified five QTLs determining daylength-independent flowering time and five QTLs determining photoperiod sensitivity. These QTLs together do not explain as much phenotypic variation as the narrow-sense heritability of the traits. Marker density is unlikely to be the cause of this discrepancy. The genetic map used for the analysis had 2677 markers with a mean distance between adjacent markers of 0.7 cM. Only one gap (of 11 cM) was larger than 7 cM. Even at this marker spacing, a large effect QTL should have been detectable. Another possible reason for such a discrepancy is the segregation of several additional minor effect QTLs for photoperiod sensitivity, which were not detected as significant; numerous QTLs for flowering have been reported for lettuce (Han et al. 2021b).

There was no overlap between the FLT and PPS QTLs, suggesting separate mechanisms. The largest QTL determining photoperiod sensitivity differed in the two SD environments; *qPPS2.1* was the most significant in the Holtville experiment, while *qPPS1.1* was the most significant in the Davis Screenhouse experiment that on average had a longer photoperiod by 30 min. This parallels experiments with Arabidopsis, in which photoperiodic regulation of flowering time differed between lab and field conditions (Song et al. 2018). This is consistent with the complex, environmentally sensitive regulatory network that determines the photoperiodic responses in flowering plants.

The largest effect flowering time QTL was *qFLT4.1*. This overlapped with a bolting time QTL, *qBLT4.1*, discovered in a F_6_ RIL population developed from a cross between a Batavia variety “Reine des Glaces” and a Latin variety “Eruption” (Mamo et al. 2019) and encompasses 280 lettuce gene models. Among them is an ortholog to Arabidopsis *FD* (Abe et al. 2005), Lsat_1_v5_gn_4_145080, and an ortholog to Arabidopsis *FLOWERING LOCUS K* (*FLK*; Lim et al. 2004), Lsat_1_v5_gn_4_138841. Both gene models carry non-synonymous variants between the coding sequences of the two parental lines (Supp. Table 2). The expression of Lsat_1_v5_gn_4_145080 in the lettuce apical meristem was quantified in Chen et al. (2018); in their study, the expression of Lsat_1_v5_gn_4_145080 reached a global maximum during the transition between vegetative growth and reproductive growth and decreased after the apical meristem committed to floral development. This is consistent with the known function of *FD* in Arabidopsis where it complexes with FT in the apical meristem to promote the floral transition (Abe et al. 2005). Armenian differs from PI251246 at five amino acid residues between positions 100 and 240. Although this region is not conserved between lettuce and Arabidopsis FD, this region is conserved across orthologs of species within the Compositae family, including lettuce, artichoke, and sunflower (Suppl. Table 4a). This does not rule out Lsat_1_v5_gn_4_145080 as a candidate for determining the phenotype of *qFLT4.1*.

In addition to *qFLT4.1*, two other flowering time QTLs collocate with the previously reported QTLs, *qFLT7.4* and *qFLT9.2*. *qFLT7.4* was first discovered in a Salinas (crisphead) × PI171674 (romaine) F_2_ mapping population (Sandoya et al. 2020). This mapping population does not share parents with the population used in our experiment. *qFLT9.2* partially collocated with a peak identified in a genome-wide association study on lettuce that investigated variations in developmental rate (Sthapit Kandel et al. 2020). There are several flowering time related orthologs within these two QTLs; however, none have non-synonymous substitutions within their coding regions. Expression level polymorphism data are not available for these lines.

Many genes encoding putative photoperiodism-related components were identified within the two largest-effect PPS QTLs. A phytochrome encoding gene, Lsat_1_v5_gn_1_41100, is located within *qPPS1.1*. Phytochromes constitute important components of the photoperiod pathway of flowering regulation in Arabidopsis (Legris et al. 2019). The phyB–phyC heterodimer is important for inhibiting flowering in non-inductive photoperiods (Monte et al. 2003; Sánchez-Lamas et al. 2016). Armenian differs from PI251246 at amino acid number 507; however, this position is not highly conserved between putative phytochrome genes in eudicot species (Suppl. Table 4b).

The other large-effect PPS QTL, *qPPS2.1*, collocates with a bolting time QTL, *qSTL2.2*, discovered in a F_7_ RIL population developed from a cross between two crisphead cvs. “Emperor” and “El Dorado” (Jenni et al. 2013). *qPPS2.1* includes a CO-like ortholog, Lsat_1_v5_gn_2_86121. The expression level of *CO* is circadianly entrained in Arabidopsis, resulting in a 24-h-phase oscillation of its transcription in vegetative tissues (Suárez-López et al. 2001). Lsat_1_v5_gn_2_86121 also shows a circadian expression pattern in vegetative lettuce leaves (Higashi et al. 2016; Fig. 8). The first non-synonymous variant between Armenian999 and PI251246 is in the 64^th^ base pair of exon 1 (Supplementary Table 2, row 143). This substitution changes a threonine in Armenian999 and the genome reference cultivar, Salinas, to proline in PI251246 at position 22 of the encoded protein. The threonine at position 22 is in the middle of the first of the two zinc fingers of CO and is conserved between the daylength sensitive genotypes of lettuce and Arabidopsis (Putterill et al. 1995; Suppl. Table 4c). Of the 17 CO-like (COL) genes in the Arabidopsis genome, only two of them, *COL5* and *COL9*, have thus far been shown to function in photoperiod control of flowering time (Cheng and Wang 2005; Hassidim et al. 2009). There are at least six COL paralogs in the lettuce genome (Han et al. 2021a); however, the functional ortholog of *CO* had not been previously identified in lettuce, despite molecular and bioinformatic efforts (Abbott 2010). Our data indicates that Lsat_1_v5_gn_2_86121 is the functional homotholog of *CO* in lettuce.

In summary, an interspecific *Lactuca* RIL population that segregates for both daylength-independent flowering time and photoperiod sensitivity provided an effective platform for studying the genetic mechanism of photoperiodic control of flowering time in lettuce. Our study showed that the photoperiodic regulation of flowering time in lettuce is distinct from genes determining daylength-independent flowering time under inductive daylength conditions. This study also revealed lettuce genes that are candidates for functional orthologs of *FD* and *CO*, key flowering time and photoperiodism regulators in Arabidopsis. The identification of the genes that fulfill these roles in lettuce has been confounded in the past due to multiple paralogs in the duplicated lettuce genome. This study provides the foundation for future experiments focused on the functional validation of these candidate genes using genome editing and transgenic complementation.

## Supplementary Information

Below is the link to the electronic supplementary material.

Supplementary file1 (TIFF 15410 KB)

Supplementary file2 (TIFF 15410 KB)

Supplementary file3 (TIFF 79109 KB)

Supplementary file4 (XLSX 153 KB)

## Data Availability

GBS data of the RILs and WGS data of the parents are available on the NCBI SRA database under BioProjects PRJNA642889, PRJNA510128, and PRJNA478460. All raw phenotype data are available in Supplementary Table 3.
