# Supplementary figures and images for: Quantitative Trait Loci and Candidate Genes Associated with Photoperiod Sensitivity in Lettuce (Lactuca spp.)
Source: Theor Appl Genet. 2021 Jul 10;134(10):3473–87. doi: 10.1007/s00122-021-03908-w (PMC8440299; doi:10.1007/s00122-021-03908-w)

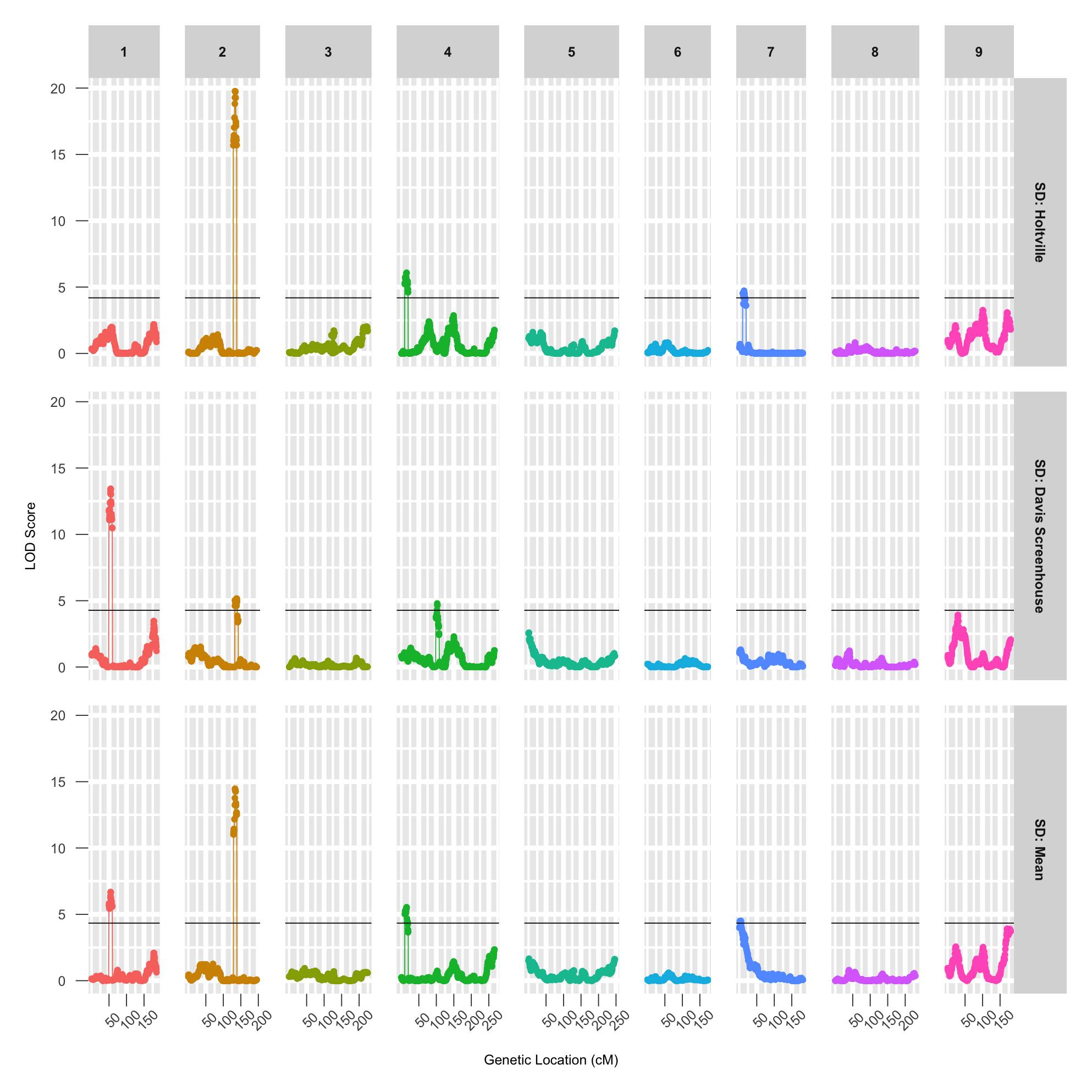

Supplement: Supplementary file 1 — Supplementary file1 (TIFF 15410 KB) [file 122_2021_3908_MOESM1_ESM.tiff]

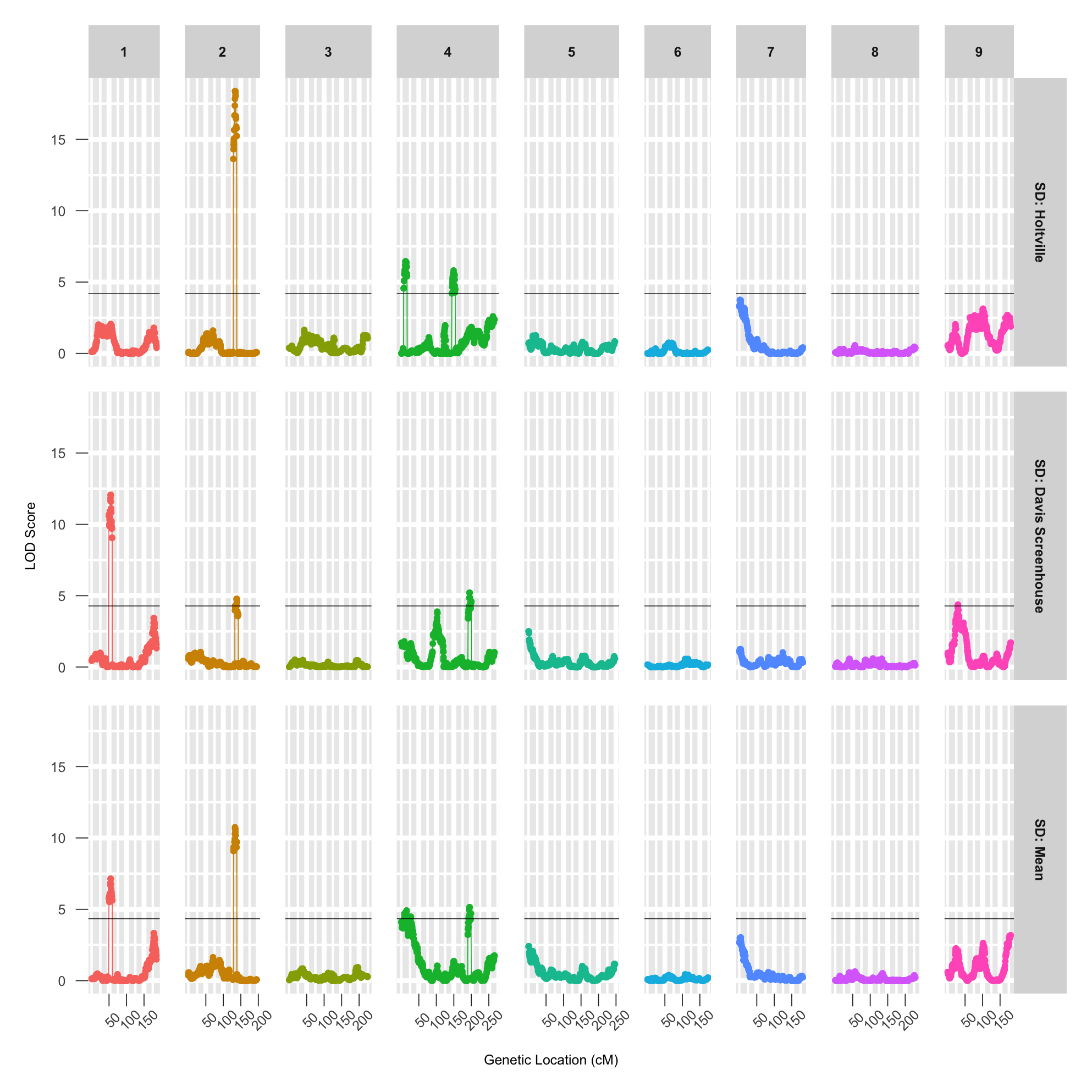

Supplement: Supplementary file 2 — Supplementary file2 (TIFF 15410 KB) [file 122_2021_3908_MOESM2_ESM.tiff]
